# Susceptibility of turkeys to pandemic-H1N1 virus by reproductive tract insemination

**DOI:** 10.1186/1743-422X-7-27

**Published:** 2010-02-03

**Authors:** Mary Pantin-Jackwood, Jamie L Wasilenko, Erica Spackman, David L Suarez, David E Swayne

**Affiliations:** 1 Exotic and Emerging Avian Viral Diseases Research Unit, Agricultural Research Service, U.S. Department of Agriculture, Athens, Georgia 30605 USA

## Abstract

The current pandemic influenza A H1N1 2009 (pH1N1) was first recognized in humans with acute respiratory diseases in April 2009 in Mexico, in swine in Canada in June, 2009 with respiratory disease, and in turkeys in Chile in June 2009 with a severe drop in egg production. Several experimental studies attempted to reproduce the disease in turkeys, but failed to produce respiratory infection in turkeys using standard inoculation routes. We demonstrated that pH1N1 virus can infect the reproductive tract of turkey hens after experimental intrauterine inoculation, causing decreased egg production. This route of exposure is realistic in modern turkey production because turkey hens are handled once a week for intrauterine insemination in order to produce fertile eggs. This understanding of virus exposure provides an improved understanding of the pathogenesis of the disease and can improve poultry husbandry to prevent disease outbreaks.

## Findings

Because of the known susceptibility of turkeys to type A influenza viruses and the history of infection with triple reassortant viruses [1-6], when the pandemic influenza A H1N1 2009 (pH1N1) emerged, the possibility of turkeys becoming infected with the novel virus was investigated. However, experimental challenge with pH1N1 virus by the respiratory route showed that both turkey poults and adult turkey hens were resistant to infection [7-9], but infection was produced in young turkeys by the novel intracloacal route of inoculation (J. Pasick, personal communication). In August 2009, pH1N1 virus was detected in two turkey breeder farms in Chile presenting drops in egg production [10]. Epidemiological investigations on the possible source of infection identified workers with respiratory problems, and hen insemination as a risk factor for virus transmission to the birds. A second and third outbreak in turkey hens occurred in Canada, in September 2009, and in the USA, in November 2009, with a marked drop in egg production as the primary clinical sign of disease [11,12]. These three outbreaks of pH1N1 influenza in turkeys raised the question of how the turkey hens became infected when experimental evidence suggested that turkeys were refractory to respiratory infection.

In our previous study, 73-week-old turkey hens and 3-week-old turkey poults were intranasally inoculated with A/Mexico/4108/09 (H1N1) [8]. None of the turkeys developed clinical signs or died, no virus was detected in tissues, and all turkeys were negative for antibodies to the virus, indicating that they did not become infected. In another study, 21- and 70-day-old meat turkeys were oro-nasally inoculated with A/Italy/2810/2009 (H1N1) influenza virus. Virus was not recovered by molecular or conventional methods from blood, tracheal and cloacal swabs, lungs, intestine or muscle tissue, and only some birds seroconverted [9]. In a third study, inoculation of 3-week-old turkeys with A/CA/07/09 (H1N1) through the intranasal and intraocular route also failed to initiate infection [7].

In order to understand how the pH1N1 virus potentially had infected turkey breeders, we conducted a study in which we inoculated 53-week-old laying turkey hens with 10^5.3 ^50% cell culture infective doses of A/Chile/3536/2009 (H1N1) virus by three different routes. Eight hens were inoculated intranasally (IN), four hens were inoculated intracloacally (IC), and four hens were inoculated through the intrauterine (IU) route. Oropharyngeal and cloacal swabs were taken from all hens at days 2, 4, 7, 10, and 14 days post-inoculation (dpi), and lung, spleen, heart, kidney and oviduct were taken from one hen per group at 3 and 7 dpi for virus detection by quantitative real-time reverse transcriptase polymerase chain reaction (qRRT-PCR) assay targeted to the influenza virus matrix gene with the described modified reverse primer 3'-cagagactggaaagtgtctttgca-5' [8,13]. Tissues were also taken for histology and viral antigen detection by immunohistochemistry (IHC). For IHC, mouse monoclonal antibody P13C11, specific for influenza A nucleoprotein, was used. Sections were stained as previously described [14]. Serum was collected from the remaining turkeys at the end of the 14-day study for antibody testing by hemagglutination inhibition (HI).

**Figure 1:**
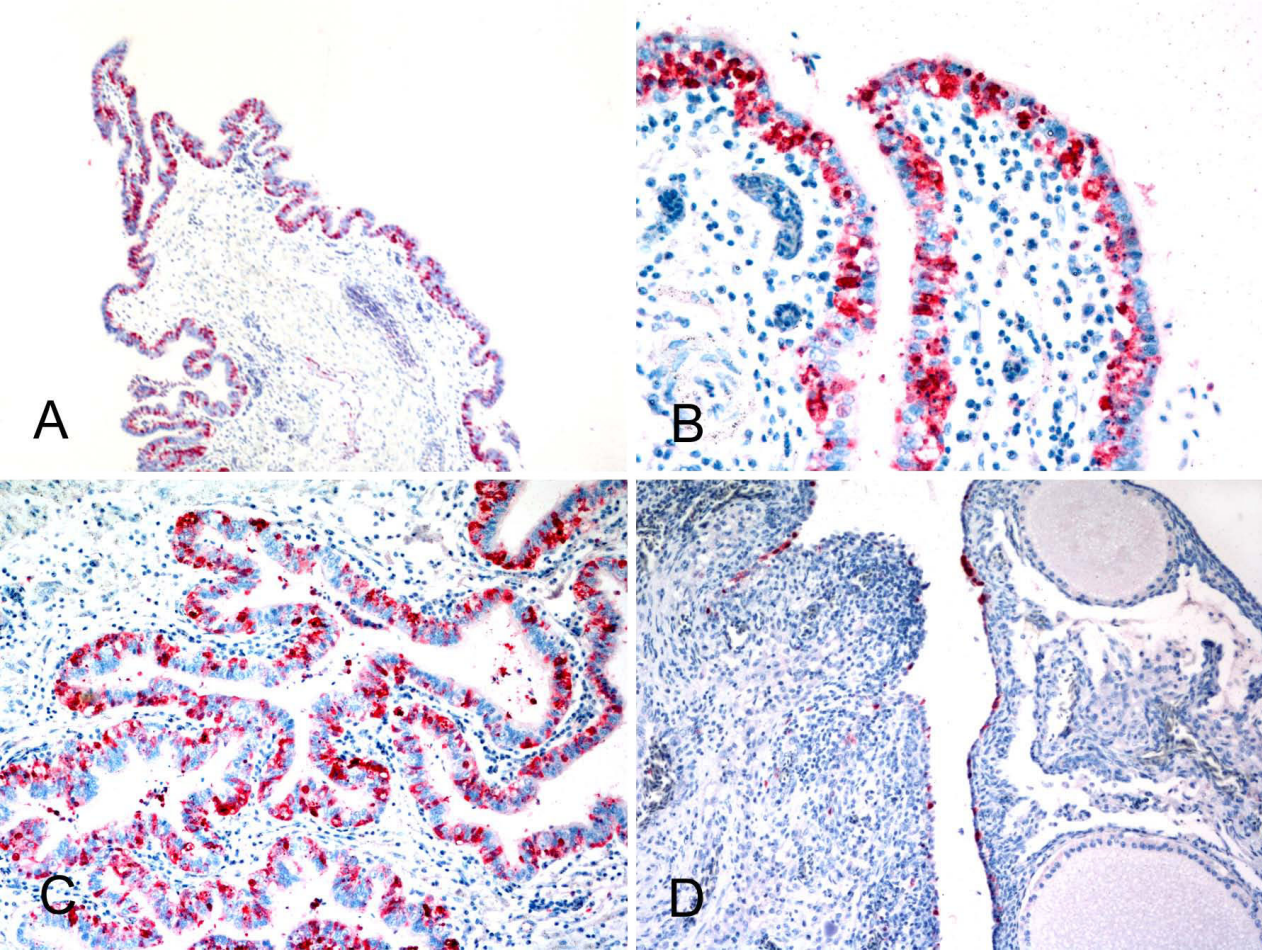
**Photomicrographs of immunohistochemically strained reproductive tracts of turkey breeder hens IU-inoculated with pH1N1 virus**. (A to C) Oviducts with influenza viral antigen in luminal lining epithelium, (D) Ovary with influenza viral antigen in surface germinal epithelium.

None of the turkeys inoculated IN with the pH1N1 virus developed clinical signs. Turkeys inoculated IC or IU presented with mild diarrhea from 1 to 4 dpi. Turkeys inoculated by the IU route stopped laying eggs at 5 dpi, while turkeys IC inoculated laid eggs daily through 9 dpi. Turkeys IN-inoculated continued laying eggs until the end of the study. Turkeys inoculated IN or IC, necropsied at 3 and 7 dpi, presented no gross lesions and had active oviducts. The oviducts of the turkeys inoculated IU were congested or undergoing involution at 3 and 7 dpi, respectively. All IN-inoculated turkeys were negative for antibodies to the virus on 14 dpi. One of two IC-inoculated turkeys had a hemagglutination inhibition (HI) geometric mean antibody titer of 256, and both hens inoculated through the IU route had high HI titers (4096 and 8192) at 14 dpi. The two hens inoculated either IC or IU and necropsied at 7 dpi also seroconverted (64 and 256 HI titers, respectively). Virus was detected in oropharyngeal and cloacal swabs from IU-inoculated turkeys from 2 to 14 dpi, and at 4 dpi from the cloacal swab of one IC-inoculated turkey (Table 1). Virus was detected in the oviduct of the turkeys IC- or IU-inoculated, and virus antigen was visualized by immunohistochemical staining in the surface germinal epithelium of the ovary and luminal epithelium lining the oviduct (Figure 1). No lesions or viral staining was present in any of the other tissues examined. No virus was detected in swabs or tissues from IN-inoculated turkeys.

**Table 1:** Results of qRRT-PCR testing for pH1N1 virus in oropharyngeal and cloacal swabs of experimental turkey hens inoculated intranasally, intracloacally, or intrauterine with A/Chile/3536/2009 (H1N1) virus.

Groups	Sampling day (days post inoculation) for swabs									
	
	2		4		7		10		14	
	
	**OP**^**a**^	**C**^**b**^	OP	C	OP	C	OP	C	OP	C
IN^c^	0/8^d^	0/8	0/7	0/7	0/7	0/7	0/6	0/6	0/6	0/6
IC^e^	0/4	1/4(10^4.7^)	0/4	0/4	0/3	0/3	0/2	0/2	0/2	0/2
IU^f^	1/4 (10^4.7^)^g^	1/4(10^6.7^)	3/3(10^4.7^)	3/3(10^5.8^)	0/3	3/3(10^5.7^)	0/2	1/2(10^5.1^)	1/2(10^4.8^)	0/2

^a ^OP, oropharyngeal. ^b ^C, cloacal. ^c ^IN, intranasal. ^d ^number of virus positive/total sampled. ^e ^IC, intracloacal. ^f ^IU, intrauterine. ^g ^average titer of RNA positive samples. Previous studies have shown correlation between qRRT-PCR results and infectious titer of influenza A virus for oropharyngeal and cloacal swabs [15]. We report our qRRT-PCR data in relative equivalent units (REU) based on a standard curve for A/Chile/3536/2009 (H1N1) in mean chicken embryo infectious doses (EID_50_)

In this study, and consistent with previous studies, turkeys IN-inoculated with the pH1N1 influenza virus did not become infected with the virus, although the respiratory route is considered the natural route of exposure for influenza A viruses in many animal species. However, IC or IU-inoculation with the virus resulted in pH1N1 virus infection. Such routes of exposure are realistic in modern turkey production because turkey hens are handled once a week for insemination, which deposits semen into the uterus, in order to produce fertile eggs, because modern tom turkeys are physically unable to efficiently breed naturally because of their large breast muscles. During this process, workers handle individual hens, manually everting the cloaca to locate the vagina for insertion of the insemination straw. Because of the close contact with infected humans, this routine insemination activity provided opportunity for initiating the infection process by either large droplet exposure during human sneezing activities or direct inoculation from infectious fomites on contaminated hands, and bird-to-bird transmission through mechanical fomite inoculation to the cloaca or reproductive tract by the inseminators. This is the first study to show infection by intrauterine exposure to influenza A virus in turkeys and such transmission is consistent with the proposed risk of infected insemination crews in cases of pH1N1 in Chilean turkey hens [10]. However, replication and shedding from the respiratory tract following IU-inoculation is perplexing considering IN-inoculation failed to produce infection. Possibly, the IU-inoculation and infection resulted in changes in the virus that allowed subsequent respiratory infection. Future studies will examine such viruses recovered from respiratory tract for changes in viral tissue tropism.

## Abbreviations

dpi: days post-inoculation; HI: hemagglutination inhibition; IC: intracloacal; IHC: immunohistochemistry; IN: intranasal; IU: intrauterine; pH1N1: influenza A H1N1 2009; qRRT-PCR: quantitative real-time reverse transcriptase polymerase chain reaction

## Competing interests

The authors declare that they have no competing interests.

## Authors' contributions

MPJ participated in the design of the study, performed the animal study, read the histopathology and immunohistochemistry slides, and drafted the manuscript. JLW conducted virus isolation and serological assays. ES carried out the qRRT-PCR studies. DLS participated in the study design. DES conceived of the study, and participated in its design and coordination, and completed the manuscript. All authors read and approved the final manuscript.
